# A Pan-Canadian narrative review on the protocols for reopening dental services during the COVID-19 pandemic

**DOI:** 10.1186/s12903-020-01340-y

**Published:** 2020-12-02

**Authors:** Mario Brondani, Denise Cua, Tala Maragha, Melody Shayanfar, Kavita Mathu-Muju, HsingChi von Bergmann, Fernanda Almeida, Jeannie Villanueva, Alexis Armando Vides Alvarado, Stephen Learey, Leeann Donnelly

**Affiliations:** 1 Department of Oral Health Sciences, Faculty of Dentistry, University of British Columbia, 116/2199 Wesbrook Mall, Vancouver, V6T 1Z3 Canada; 2 Department of Oral Biological and Medical Sciences, Faculty of Dentistry, University of British Columbia, Vancouver, Canada; 3 Department of Psychology, Faculty of Science, University of British Columbia, Vancouver, Canada; 4 MidMain Community Health Center, Vancouver, Canada; 5 Vancouver Aboriginal Health Society, Vancouver, Canada; 6 Strathcona Health Society, Vancouver, Canada

**Keywords:** Oral health care, Protocols, COVID-19, SARS-CoV-2, Narrative review, Evidence-based, Canada

## Abstract

The current coronavirus disease 2019 (COVID-19) pandemic is impacting the way in which dental services are provided. The aim of this narrative review was twofold: to summarize key areas from the Canadian protocols available for the reopening and restructuring of dental services across the country and to critically review these protocols based on existing evidence. A narrative review of the existing Canadian protocols, written in English and French, was undertaken between April 15 and July 13, 2020. The protocols were obtained by searching through regulatory bodies and websites from professional organizations, and from personal contacts through academic institutions and policy leaders. The data extraction form focused only on protocols related to dentistry, and the information was compiled by a hired assistant. Content was categorized via group discussions with the research team on eight areas: office management and procedures, patient and staff screening, treatment procedures, office layout, risk reduction, personal protective equipment, supporting information, and length and readability. Thirteen protocols were identified and offered substantial variation in the level of details provided. All but two protocols specified proper donning/doffing of personal protective equipment, while all protocols recommended daily monitoring of COVID-19 related signs and symptoms in staff and patients. They varied in terms of recommended mask types, eye and face shield protection, and head coverings. While all protocols aimed at restructuring emergency dental services, their recommendations were often not based on the published evidence. This narrative review summarized key areas from 13 provincial and territorial protocols in Canada to help oral health care providers plan the reopening of their services. The information conveyed across all documents was clear, but variance highlights the need for a coordinated effort to develop an evidence-based document for dental practitioners.

## Background

The World Health Organization (WHO) declared the novel coronavirus disease 2019 (COVID-19) a global pandemic on March 11, 2020. The disease is caused by severe acute respiratory syndrome coronavirus 2 (SARS-CoV-2), first identified in Wuhan, China in December 2019 [1, 2]. As of October 10, 2020, COVID-19 has spread to more than 200 countries and territories, caused over 1 million deaths and infected more than 35 million people globally. The pandemic also caused an unprecedented global financial crisis and recession [3], as most countries imposed internal lockdown measures, halted industrial and commercial production, and implemented border closures that caused severe disruptions to public and private services; these actions were implemented in order to minimize the spread of SARS-CoV-2 infections. One of the services greatly impacted by the pandemic has been the provision of oral health care, due to the close face-to-face proximity of dental professionals to patients and the known fact that the virus causing COVID-19 can be found in saliva droplets and aerosols – both of which are inevitable in dental procedures [1, 2, 4]. Therefore, the practice of dentistry is said to be at the highest risk for the transmission of SARS-CoV-2 [5, 6].

Although appropriate personal protective equipment (PPE) and office management and procedures are already integral components of dental care required to mitigate the risks of pathogen transmission, the decision was made to either discontinue elective procedures and attend to dental emergencies only or to close a dental practice altogether [7, 8]. The focus on emergency-only care was encouraged by the British Columbia Dental Association on March 16, 2020. Under the current pandemic reopening plan, an approach has been implemented to restructure the delivery of oral health care across Canada. Part of this restructuring includes the implementation of recommendations put forward by national and provincial governments and professional organizations in the form of guidelines, referred to herein as protocols. As suggested by Cantore and Ballini [9], dental providers should follow a firm protocol when delivering care. As different Canadian provinces and territories varied in the way they handled lockdowns, implemented testing, and curtailed general services, different levels of SARS-CoV-2 infection rates and COVID-19 development were observed, with Quebec showing more than 10,000 COVID-19 cases per 1 million population, and New Brunswick showing less than 300 COVID-19 cases per 1 million population (as of October 10, 2020). Similar variance was observed in different countries around the world, with the United States, Qatar, and Brazil showing more than 20,000 COVID-19 cases per 1 million population, and with New Zealand, South Korea, and Japan showing less than 1000 COVID-19 cases per 1 million population (infection rates refer to October 10, 2020) [10]. The extent to which these variations influenced the content of the Canadian protocols remains unknown. Uncertainties also exist among dental professionals on how to provide care during the COVID-19 pandemic [11]. In response to the need for guidance, this narrative review critically assessed the available Canadian provincial and territorial protocols in terms of content and the use of evidence-based information to better advise oral health care regulatory bodies, professionals, and decision makers.

The aim of this narrative review was twofold: to summarize key messages on the reopening and restructuring of dental services across Canada and to critically review those protocols based on existing evidence. This study is part of a larger research project entitled “Structural preparedness during the COVID-19 pandemic and the provision of urgent oral health care” aimed to determining what preparations during an outbreak are necessary to provide oral heath care, how much guidance is given for these preparations, and the barriers and facilitators involved in these preparations.

## Methods

A narrative review of the existing Canadian protocols regarding requirements for the reopening of oral health care services was undertaken between April 15 and July 13, 2020. Only protocols focusing on the practice of general dentistry were sought, both in English and French. Protocols aimed at restructuring the practice of dental hygienists, dental therapists, dental technicians, and denturists were excluded. Protocols focused on restructuring the practice of any of the dental specialties were also excluded. In addition, this narrative review excluded international guidelines for restructuring the practice of dentistry, even if they aided, in full or in part, in the development of any of the Canadian protocols. These protocols were excluded in order to offer a more focused critique using Canada as the context and to purposefully select a manageable number of protocols to be reviewed in a timely manner, given the urgency brought on by the pandemic. Within this narrative review, we aimed to look at different aspects of the protocols as described herein, identify areas of variance, and explore the extent to which evidence is being utilized (or not) to inform recommendations and guidance; the available evidence in these areas can be applicable to any jurisdiction or country when dealing with this novel coronavirus.

Given the nature of a protocol as an official governing affairs’ document outlining professional procedures related to actions and treatments, it was likely that they would be available within the grey literature and not within search engines such as PubMed^®^ (via the National Center for Biotechnology Information). Therefore, a search was conducted via Google Scholar, mailing lists from regulatory bodies, the websites of organized professions and the government, and personal contacts through academic institutions and dental policy leaders in Canada.

Data extraction was performed by a hired research assistant (DC) with content being categorized during group discussions with members of the research team for consensus. Each protocol was assessed using a data extraction form developed by MB, LD, and DC based on the available literature, and included the following eight areas: office management and procedures (e.g., cleaning and disinfection), personal protective equipment (e.g., masks, facial shield, eye protection), patient and staff screening (e.g., signs and symptoms of COVID-19), transmission risk reduction (e.g., used of pre-procedural rinse, consulting a heating, ventilation, and air conditioning professional), procedures (e.g., types of clinical and non-clinical treatment the protocol is aimed for), supporting information (e.g., the recommendations are supported by citations), office layout (e.g., patient and staff flow, barriers), and length and readability (e.g., clarity, easiness to read). These eight areas were also used to structure the analysis of the protocols and present the findings. It was required that the protocols contain guidance on resuming dental care following closure of the services, or restructuring of dental care beyond emergencies, due to the COVID-19 outbreak. The protocols must also have focused on minimizing SARS-CoV-2 transmission and infection and needed to have been published or updated as of July 13, 2020. However, with the evolving nature of the COVID-19 pandemic on its second and more severe wave of infections, many protocols are continuously being updated; therefore, the information provided herein is subject to change.

## Results

A total of 15 different protocols were found; however, only 13 were utilized here (Table 1), with each Canadian province and territory having their own protocol devised by the following groups: the Alberta Dental Association and College, the College of Dental Surgeons of British Columbia, the Manitoba Dental Association, the Royal College of Dental Surgeons of Ontario, the Dental Association of Prince Edward Island, the Ministère de la Santé et des Services Sociaux and the Université de Montréal for Quebec, the College of Dental Surgeons of Saskatchewan, the New Brunswick Dental Society, the Provincial Dental Board of Nova Scotia, the Newfoundland and Labrador Dental Board, the Dental Services from the Department of Health of the Government of Nunavut, the Government of the Northwest Territories, and the Government of the Yukon. Two additional protocols were found in British Columbia, but were not included in the analysis as they were later replaced by the College of Dental Surgeons of British Columbia protocol. We have summarized the recommendations for the eight areas found in each protocol (Table 1) (protocols can be found at https://www.diac.ca/wp-content/uploads/2020/05/National-Reopen-Strategy-.pdf).

**Table 1:** Canadian protocols for the reopening and restructuring of dental services across different provinces and territories according to office management and procedures, patient and staff screening, treatment procedures, office layout, personal protective equipment, supporting information, and length and readability

		British Columbia	Alberta	Saskatchewan	Manitoba	Ontario	Nova Scotia	New Brunswick	Newfoundland and Labrador	Prince Edward Island	Yukon	Nunavut	Quebec	Northwest Territories
		May 15, 2020	June 12, 2020	July 13, 2020	May 22, 2020	May 31, 2020	July 3, 2020	Date N/A	June 23, 2020	June 12, 2020	June 1, 2020	June 5, 2020	May 28, 2020	June 19, 2020
*Office management and procedures*^a^														
Minimize access points		✓	N/S	N/S	✓	✓	N/S	N/S	N/S	N/S	N/S	N/S	N/S	✓
Remove non-essential items		✓	✓	✓	✓	✓	✓	✓	✓	✓	✓	✓	✓	✓
Patient wait in car/outside		N/S	N/S	N/S	N/S	✓	✓	✓	N/S	✓	N/S	N/S	✓	N/S
Signage upon entry/admission		✓	Consider	✓	✓	✓	N/S	✓	✓	N/S	✓	✓	✓	✓
Patient hand hygiene		✓	✓	✓	✓	✓	✓	✓	✓	✓	✓	✓	✓	✓
Patient face covering		N/S	N/S	✓	✓	✓	N/S	✓	N/S	Consider	✓	✓	✓	Advise Patient
Limit number of staff		✓	N/S	✓	N/S	✓	N/S	✓	N/S	✓	✓	✓	✓	✓
Limit number of people inside		✓	✓	✓	✓	N/S	N/S	✓	✓	✓	✓	✓	✓	✓
Stagger staff and patient schedule		✓	✓	✓	N/S	✓	✓	✓	✓	✓	✓	✓	✓	✓
High-touch surfaces		Clean regularly	Clean and Disinfect 2x/daily	Clean 2x/day	Sanitize frequently	Clean and disinfect 2x/day	Disinfect regularly	Clean and Disinfect frequently	Disinfect 2x/day	Clean 2x/day	Clean 2x/day	Clean and Disinfect 2x/day	Clean daily	Clean and Disinfect 2x/day
Water lines		Flush 20–30 secs (before and b/w pts)	Shock after extended break	N/S	N/S	Shock after extended break	Flush 2 min (start of day) and 30 secs (after pts)	N/S	Shock after extended break	N/S	Shock after extended break	Shock at start of each clinic visit	Flush 2 min (start and end of day)	Shock after extended break
*Patient and staff screening*^c^														
Patient screening		✓	✓	✓	✓	✓	✓	✓	✓	✓	✓	✓	✓	✓
Staff screening		✓	✓	✓	✓	✓	✓	✓	✓	✓	✓	✓	✓	✓
*Treatment procedures*^e^														
Patient risk assessment		✓	✓	✓	✓	N/S	N/S	✓	✓	✓	✓	✓	✓	✓
Pre-existing health conditions		✓	✓	N/S	N/S	N/S	N/S	✓	✓	✓	N/S	✓	N/S	✓
Age		> 70	> 65	Seniors	Older person	N/S	N/S	> 65	> 60	> 60	N/S	> 60	N/S	N/S
Emergency		✓	✓	✓	✓	✓	✓	✓	✓	✓	✓	✓	✓	✓
Urgent		✓	✓	✓	✓	✓	✓	✓	✓	✓	✓	✓	✓	✓
Non-essential		✓	✓	✓	N/S	✓	✓	✓	✓	✓	✓	✓	✓	✓
For C-19 + or suspect patients		✓	✓	No	No	✓	No	No	No	No	✓	✓	✓	No
*Office layout*^g^														
Rearrange office for distancing		✓	✓	✓	✓	N/S	N/S	✓	✓	✓	✓	✓	✓	✓
Enclosed AGP room		N/S	N/S	✓	N/S	✓	N/S	N/S	N/S	N/S	✓	✓	✓	✓
Specified donning/doffing areas		✓	N/S	✓	N/S	N/S	N/S	✓	N/S	N/S	N/S	N/S	N/S	✓
*Personal protective equipment*^b^														
NAGP														
Mask type and ASTM level		Surgical 2/3	Surgical 2/3	Surgical 2/3	Surgical 2/3	Surgical 2/3	Surgical level N/S	Surgical 3	Surgical 2/3	Surgical 2/3	Surgical 2/3	Surgical 2/3	Surgical 2 or higher	Surgical 2/3
Eye protect		✓	OR	✓	OR	OR	AND/OR	✓	OR	OR	OR	OR	OR	OR
Face shield		N/S		N/S				N/S						
Office attire		✓	✓	✓	When possible	✓	✓	✓	✓	✓	✓	✓	✓	✓
Gown/lab coat		N/S	N/S	N/S	N/S	N/S	N/S	N/S	N/S	✓	✓	✓	✓	✓
AGP														
Mask type and ASTM level		N95	N95	N95/KN95	N95	N95	N95	N95	N95	N95	N95	N95	N95	N95
		OR	OR	OR	OR	OR	OR	OR	OR	OR	OR	OR		
		Surgical 3 + FS	Surgical 3 + FS	Surgical 2/3 + FS	Surgical 3 + FS	Surgical 2/3 + eye protection and/or FS	Surgical Level N/S + FS	Surgical 3	Surgical 2/3 + FS	Surgical 3	Surgical 2/3	Surgical 2/3		
Eye protect		OR	OR	AND/OR	OR	AND/OR	AND/OR	✓	✓	OR	OR	AND/OR	N/S	OR
Face Shield								✓	✓				✓	
Office attire		✓	✓	N/S	When possible	✓	✓	✓	✓	✓	✓	✓	✓	✓
Gown/lab coat		✓	✓	✓	✓	✓	✓	✓	✓	✓	✓	✓	✓	✓
Head cover		✓	✓	✓	✓	N/S	N/S	✓	N/S	✓	✓	N/S	N/S	✓
Booties		✓	N/S	N/S	NS	✓	N/S	N/S	✓	N/S	N/S	N/S	N/S	N/S
*Transmission risk reduction*^d^														
Proper donning/doffing		✓	✓	✓	N/S	✓	✓	✓	✓	✓	N/S	✓	✓	✓
Pre-procedural rinse (seconds)		N/S	1% HO_2_ (30)	1% HO_2_ (60)	HO_2_	1–1.5% HO_2_ OR 1% PI (60)	1% HO_2_ OR 0.5–2% PI (min 30)	1–1.5% HO_2_ (30–60)	1–1.5% HO_2_ (30–60) optional	1% HO_2_ (60)	N/S	1–1.5% HO_2_ OR 1% PI (60)	Antiseptic (60 or 2 × 30)	1% HO_2_ (60)
Rubber dam		✓	✓	✓	✓	✓	✓	✓	✓	✓	N/S	✓	✓	✓
HVE		✓	✓	✓	✓	✓	✓	✓	✓	✓	N/S	✓	✓	✓
4-handed dentistry		N/S	✓	✓	✓	NS	✓	✓	✓	✓	N/S	N/S	N/S	✓
Minimize AGP		✓	✓	✓	✓	✓	N/S	N/S	N/S	✓	✓	✓	✓	✓
Minimize intra-oral imaging		✓	N/S	✓	✓	✓	N/S	✓	✓	✓	N/S	✓	N/S	✓
HVAC/air filtration unit		✓	N/S	✓	N/S	Consider	N/S	N/S	Rec’d	N/S	✓	✓	✓	✓
Professional consult for HVAC		✓	N/S	✓	N/S	✓	N/S	✓	✓	N/S	✓	✓	✓	✓
Settling time b/w pts (min)		N/S	N/S	15	N/S	15	Not required	N/S	15	10	N/S	15	N/S	15
*Supportive information*^f^														
CDC or provincial health officers and ministries		✓	✓	✓	✓	✓	✓	✓	✓	N/S	✓	✓	✓	✓
WHO		N/S	✓	N/S	✓	✓	✓	N/S	N/S	N/S	N/S	N/S	✓	✓
Occupational Safety Organizations		Worksafe	✓	N/S	N/S	N/S	N/S	Worksafe	CCOHS, NIOSH	CCOHS	N/S	CCOHS	WorkSafe	OCPHO
														NIOSH
Length and readability^h^														
Total number of pages		19	15	10	8	16	16	12	25	23	7	85	60	15
> 75% text		✓	✓	✓	✓	✓	✓	✓	✓	✓	✓	✓	No	✓
PPE/procedure table		N/A	✓	✓	N/A	✓	✓	N/A	N/A	✓	✓	✓	✓	✓
Appendices/resources		Links	Links	✓	N/A	Links	Links	Links	Links	Links	N/A	✓	Imbedded	Imbedded
Printable resources		N/A	✓	N/A	✓	✓	✓	✓	✓	✓	N/A	✓	✓	N/A
References		N/A	N/A	N/A	✓	✓	✓	N/A	N/A	✓	✓		✓	✓
Others		Updates highlighted		Updates highlighted Definitions provided				Screening Questions and posters (hand hygiene, physical distancing)	Glossary of terms	Decision Trees Glossary of Terms and Acronyms	Refers readers to Alberta Dental Assoc. and College for AGP	IPC Manual	Tips and Tricks Lab area Teaching environment In home care Definitions List of Emerg clinics	

*N/C* no changes, *N/A* not available, *N/S* not specified, *AGP* aerosol generating procedures, *NAGP* non-aerosol generating procedures, *G* gown, *LC* lab coat, *FS* face shield, *ASTM* American Society for Testing and Materials, *CDC* Centers for Disease Control and Prevention, *WHO* World Health Organization, *HVE* high volume equipment, *HVAC* heating, ventilation, and air conditioning, *PI* Povidone-iodine, *Rec’d* recommended, *CCOHS* Canadian Centre for Occupational Health and Safety, *NIOSH* National Institute for Occupational Safety and Health (United States of America), *IPC* Infection Prevention and Control Professional

^a^OPE—Operatory protective equipment: refers mostly to equipment disinfection, functionality and maintenance including, but not limited to: use of disposable protective surface barriers, identification of the level of contaminants, scheduling of equipment and unit testing, routine check on medical supplies expiry dates, water line flush, storage of unnecessary items, and so on

^b^PPE—Personal protective equipment: Refers to the use of surgical masks (level 1, 2 and 3) and/or respirators (N95/99/100), gowns, lab coats, gloves, eye protection (e.g., Google), facial protection (e.g., facial shield), or a combination of, or other (specified)

^c^Refers to the screening of staff and patients for risk factors (close personal contact with a suspected or lab confirmed COVID-19 individual within the last 2 weeks, travel outside of the province or country in the previous 2 weeks) and symptoms (fever > 38C, cough, sore throat, shortness of breath, loss of the sense of smell, Flu-like symptoms, runny nose). Some protocols provided the actual form for the intake of this information

^d^Refers to procedures and interventions aimed at preventing or eliminating the transmission of SARS-CoV-2 including proper donning/doffing, use of pre-procedural rinse, use of rubber damn, high volume suction unit, air filtration, and so on

^e^Refers to the description of treatment procedures that the protocols are aimed for, from emergency to preventive, curative, surgical, and other treatment

^f^Refers to source of information used to develop the protocol, including expert opinion, and evidence-based documents

^g^Refers to specific recommendations for the layout of the clinic (e.g., waiting area space, front desk physical separation, and so on), and the operatory (e.g., negative/positive pressure room, floor-to-ceiling enclosure, and others)

^h^Refers to the way the information is presented in terms of clarity and comprehension, use of infographics (tables, figures, schematics), easiness to follow, and so on

### Office management and procedures

All protocols highlighted cleaning and disinfection recommendations following treatment of patients during the COVID-19 pandemic, with increased or enhanced routine cleaning already in use in dental practices. All protocols also stipulated the removal of non-essential items from waiting areas and across the office, and about informing patients on hand hygiene. Five protocols recommended minimizing access points, while signage related to COVID-19 and the appropriate precautions was recommended in 10 protocols. The majority of protocols recommended that physical distancing measures be adhered to, and that the number of people inside the practice be limited (n = 11 protocols). Six protocols recommended that water lines be shocked after an extended break, while eight protocols recommended cleaning and/or disinfecting high-touch surface areas at least twice a day.

### Patient and staff screening

All 13 protocols recommended staff and patients be screened for known symptoms of COVID-19, including fever and cough; preferably, patients should be screened over the phone prior to the appointment and re-screened at the appointment date. Numerous protocols also appended the screening question form or provided a link to one. All protocols also had provisions that staff be provided with training on COVID-19 screening and risk transmission reduction.

### Treatment procedures

All 13 protocols outlined the scope and intent of their recommendations for urgent and emergency care, while only one protocol did not specify if the recommendations would also apply to non-essential care. At least six protocols referred specifically to the need for risk assessment for the provision of oral health care to COVID-19-positive, or suspected positive, patients. Eight protocols focused on the age of older adults considered for risk assessment, with seven focusing on pre-existing conditions as part of this assessment, rather than age alone. The majority of protocols suggested booking appointments earlier in the day or on a separate day for known high-risk populations including seniors and those immune compromised.

### Office layout

The office layout, from waiting areas to operatory and sterilization rooms, had the widest variation in terms of recommendations. All protocols recommended removing non-essential items (e.g., magazines, wall frames) from wherever possible in the office; however, only four protocols recommended designated areas for donning and doffing of personal protective equipment. In addition, seven protocols did not appear to recommend a specific closed operatory for aerosol-generating procedures.

### Personal protective equipment (PPE)

All protocols recommend PPE as regular components of the provision of any dental care, including aerosol-generating (AGPs) and non-aerosol generating (NAGPs) procedures. However, they varied in terms of recommended mask types, eye and face shield protection, and head coverings. For AGPs, two protocols seemed to recommend both eye and face shield protection, while five advocated use of one or the other, but not both. ASTM (American Society for Testing and Materials) Level 2 or 3 surgical masks were suggested for NAGPs in all protocols, while six protocols recommended a N95 respirator or a Level 3 surgical mask and a face shield for AGPs. None of the protocols explicitly mentioned the use of negative pressure rooms for the management of aerosols and air circulation, nor did they refer to national clinical waste regulations or decontamination policies.

### Transmission risk reduction

Eleven protocols recommended the use of a pre-procedural rinse for all patients, while eight specified various durations and concentrations of hydroperoxyl radical (HO_2_) as the actual rinsing solution to be used. Additionally, 12 protocols recommended the use of rubber dams and high-volume suction as frequently as possible. Eight protocols recommended consulting a heating, ventilation, and air conditioning (HVAC) professional to help determine the air changes per hour that could be utilized to calculate the settle time after AGPs with COVID-19-positive, or suspected positive, patients. Six protocols specified the amount of time needed for aerosol droplets to settle prior to disinfection of the room to reduce the potential for virus transmission. The use of four-handed dentistry was supported by eight protocols.

### Supporting information

Most protocols (n = 12 protocols) appeared to be developed from professional agency recommendations (e.g., the Center for Disease Control and Prevention (CDC) and/or the WHO). The names of the authors were outlined in one protocol (from Quebec), while the others had the issuing organization as the author. None of the protocols indicated patient representation or involvement during the development process.

### Length and readability

The lengths of the protocols were between 7 and 60 pages, with all but one having more than 75% pure text (excluding appendices). Some inconsistency in information was found within the same protocol. For example, when referring to the use of various mask levels and types for the same procedure, the same protocol mentioned “and/or”, then just “and”, and later suggested just “or”. Eleven protocols provided appendices or other resources to help with the implementation of their recommendations. One protocol also included useful “tips and tricks” and a list of all clinics providing emergency care for COVID-19-positive (confirmed), or suspected positive, patients.

Numerous protocols seemed to differentiate their recommendations based on the SARS-CoV-2 infection status of the patient. If patients were positive for SARS-CoV-2 (confirmed), one protocol suggested they wait in the car or outside, two recommended using an enclosed operatory for AGPs during treatment, six endorsed the use of N95 (or higher) masks for any procedure performed, two preferred the use of a pre-procedural rinse and rubber dam, and two recommended minimizing the generation of aerosols and the need for intra-oral radiography (data not shown in Table 1).

## Discussion

Our study sought to critically review and summarize eight key areas of the 13 protocols available in Canada for the reopening and restructuring of oral health care services across different provinces and territories. All protocols had similar recommendations in some areas (e.g., increased or enhanced routine cleaning, screening staff and patients for known symptoms of COVID-19, and use of PPE as regular components of the provision of any dental care) yet differed in others (e.g., risk assessment, face and head protection, types of pre-procedural rinses to be used). Although variation in the provision of services is related to a dentist’s personality and philosophy of care, the features of the practice, and patients’ preferences [12, 13], protocols are necessary for the profession and its services [14]—particularly during this unprecedented pandemic.

This narrative review of the 13 protocols showed that ‘office management and procedures’ was not a term used explicitly, but rather it was implied when referring to waterline maintenance and disinfection of high-touch surfaces in the operatory rooms as part of the current daily routine in a dental office [15]. In the era of COVID-19, at least six protocols suggested increasing the frequency of this routine to effectively minimize cross-contamination [16, 17]. Across protocols, there was variation in the way the words “clean”, “sanitize” and “disinfect” were used when referring to high-touch surfaces; sanitization and disinfection are different and sanitization alone normally does not refer to eliminating viruses. Such variation might reflect the lack of consistency in which these terms are used in the literature pertaining to health care, with “clean and disinfect” as one encompassing term [18, 19], “clean/disinfecting” as interchangeable terms [20], and “cleaning” [21] or “disinfection” [22] used separately. Regardless of the term used, all protocols promoted patient safety and the prevention of the spread of pathogens.

All protocols suggested daily screening of staff for COVID-19 symptoms and screening for patients at the time of booking, as well as upon arrival at the office for in-person care. The screening revolves around identifying potential risk factors and COVID-19 symptoms, including fever [23]. In terms of fever in particular, all protocols referred to 38 °C or above, but were not specific on how to measure this temperature (e.g., oral–sublingual, forehead–temporal artery, etc.); even thought a patient is considered febrile if the oral–sublingual temperature is above 37.5 °C and despite the fluctuations in internal body temperature regulating daily circadian rhythms [24]. Some protocols provided the actual information intake form, while others added to the current patient intake form. None of the protocols suggested using point-of-care diagnostic tests (via a nasopharyngeal swab for use with polymerase chain reaction-PCR-assays to detect viral RNA) or screening (via blood samples, to detect antibodies against SARS-CoV-2). Although other point-of-care tests are available to oral health care providers [25, 26], there is currently a lack of a gold standard to confirm positive COVID-19 cases [27] given that PCR tests seem to have a high false-negative rate [28, 29]. Nonetheless, SARS-CoV-2 diagnostic tools are continually being developed and tested to curtail widespread infection and its fatal respiratory complications [30].

PPE is included in the principles of universal, or standard, precautions for infection prevention and control in a dental practice, which treats every patient and their bodily fluids as infectious for any blood-borne pathogen, even when the patient is unaware of the infection or is asymptomatic [31]. PPE became mainstream in dentistry after the HIV epidemic in the 1980s [32]. As a universal precaution, the assumption is that all patients are treated equally without instigating perceived prejudice. Although HIV and SARS-CoV-2 have very different routes of transmission, Alharbi and colleagues suggest considering “*every patient as a potential asymptomatic COVID-19 carrier*” [33], so that proper precautions are always followed. As such, it was surprising to notice that a number of protocols described procedures or equipment that was only to be used with COVID-19-positive patients (symptom or laboratory confirmed) or they referred these patients to a hospital. Since as many as 50% of SARS-CoV-2 infections remain asymptomatic [34, 35] and the percentage of false-negative results vary dramatically [36, 37], we urge caution in approaching COVID-19-positive patients differently than their allegedly negative counterparts, given the potential for fear and discrimination [11].

Mask recommendations were consistent for NAGPs; however, there was variation when it came to AGPs. Many of the protocols that referenced the WHO recommendations for PPE mentioned use of a surgical ASTM Level 2 or 3 mask or an N95 respirator or its equivalent; however, two protocols only recommended N95 masks. Among the protocols that also included information about AGPs involving COVID-19-positive (confirmed or presumed), or suspected positive, patients, a N95 respirator or equivalent was recommended. However, current evidence shows that this may be an uncomfortable and unnecessary practice given that these masks are not found to be superior to surgical masks at preventing most viral respiratory infections [38]; however, research should now focus on the efficacy of other PPE (eye protection, facial shields, and clothing) in preventing the spread of SARS-CoV-2.

Risk-reduction measures for transmission were quite comprehensive and similar across most protocols. However, not all protocols specified the need to use four-handed dentistry, employ air filtration, minimize intraoral radiographic imaging, use a particular pre-procedural mouth rinse, and monitor operatory settle times between patients. The four-handed technique has been part of almost all dental procedures, and is also believed to be beneficial for controlling SARS-CoV-2 infection [39]. None of the protocols suggested the use of negative-pressure rooms to help prevent airborne infectious particles from escaping the operatory into corridors and other rooms, as used in hospitals [40]. The implementation of negative-pressure rooms in dentistry seems to be a drastic and expensive approach for most general dental practices [41, 42]. Alternatively, AGP rooms with floor to ceiling walls (or the equivalent), good air filtration (e.g., HVAC systems), and sufficient air circulation have previously been suggested for the practice of dentistry [43]; these suggestions become especially important during the COVID-19 pandemic [44]. Six protocols did not specifically mention either air filtration or circulation measures.

Six protocols specified 10 or 15 min as the amount of time needed for aerosol droplets to settle prior to disinfection of the room to reduce the potential for virus transmission, while the others did not specify a time. In a recent statement, the American Dental Association Task Force on Dental Practice Recovery stated that “*while there is no strong evidence that supports a one-size-fits-all 15-min waiting period recommendation, it’s still very important to allow some time for aerosol droplets to settle prior to disinfection of the room to reduce the potential for virus transmission … dependent on a number of variables based on the individual practice setting*.” [45] Given the emerging evidence of asymptomatic transmission of SARS-CoV-2, this is a particularly important area of research to ensure that guidelines for environmental controls in dental offices are realistic, effective, and consistent [46]. None of the protocols seemed to differentiate between settling time as “*the amount of waiting time needed from dismissing the patient to starting to disinfect the operatory room*” or as “*the amount of waiting time needed in-between patients*”; this time is highly dependent on air circulation, air filtration, the amount of aerosols generated, the COVID-19 status of the patient, and so on.

As throat and salivary glands are likely sites for the replication and transmission of SARS-CoV-2 [47], O’Donnell and colleagues reviewed the literature on the use of mouth rinsing agents to advocate for more research on their use in reducing transmission of the novel coronavirus [48]. Although there is limited research on mouth rinses and SARS-CoV-2, eleven protocols advocated the pre-procedural use of HO_2_ (the hydroperoxyl radical, which is the protonated form of superoxide), povidone-iodine, or an antiseptic using a variety of concentrations and rinsing times. While some evidence exists that povidone-iodine may be superior in inactivating SARS-CoV-2 in vitro [49], more research is needed in this area to assess the in vivo effectiveness of pre-procedural rinses—as the virus is also shed in saliva which continues to be produced after the rinse. Our narrative review does recognize mouth rinses as useful adjuncts to plaque-control during oral hygiene, with various formulations ranging from alcohol-based chemical agents, chlorhexidine, cetylpyridinium chloride, and triclosan [50, 51].

At least nine protocols favoured the use of minimal and less invasive dental procedures that tend to minimize or eliminate the generation of aerosols. Such procedures could involve silver diamine fluoride therapies [52, 53] and atraumatic restoration techniques [54]. In fact, the COVID-19 pandemic might bring about a silver-lining moment for the profession, where there is an opportunity to favour, and be an advocate for, less invasive and conservative treatments, along with increasing the use of tele-dentistry [55].

All protocols were specific about their recommendations focused on both emergency and elective care; however, one did not specify if the recommendations would also apply to non-urgent or non-essential care. Although non-essential or elective oral health care treatments were strongly discouraged in Canada following the declaration of COVID-19 as a pandemic in March 2020 [56], they are included in 12 of the protocols, with 10 favouring NAGPs during the pandemic as suggested by Eden and colleagues [57]. In general, dental procedures are usually categorized as elective procedures (e.g., cosmetic dentistry), non-urgent procedures (e.g., replacement of non-decayed yet defective restorations), urgent conditions that can be managed with minimally invasive procedures and without aerosol generation (e.g., surgical postoperative dry socket dressing changes), urgent conditions that need to be managed with invasive and/or aerosol-generating procedures (e.g., extensive caries or defective restorations causing pain), and emergency management of life-threatening conditions (e.g., trauma involving facial bones that potentially compromises the patient’s airway) [21, 58]. Although the protocols did not use these definitions and/or exemplifications for the types of treatment, they likely meant to cover them in the restructuring of oral health care services in Canada beyond emergencies only. However, recent WHO guidance from August 3, 2020 appears to recommend postponing non-urgent and elective dental care [59] after citing a 2004 review article of in vitro studies showing that viruses may be present in dental instruments used in AGPs [60]. Such recommendations have to be taken with caution and, as suggested by Goldman, “*a more balanced perspective is needed to curb excesses that become counterproductive*” [61]. Routine and preventive care remain necessary for the early detection and control of oral diseases during the COVID-19 pandemic [62]. While booking appointments for seniors and those with underlying health conditions earlier in the day is meant to protect known high-risk populations, none of the protocols set priorities for testing patients suspected of having COVID-19 [30].

Office layout suggestions were quite consistent across the protocols regarding the need to facilitate physical distancing; however, less than half of the protocols specified the need for an AGP room or specific PPE donning and doffing areas. Some protocols may not have included these two important aspects because they were recommending minimal use of AGPs and/or not providing care to COVID-19-positive patients (confirmed or presumed). In addition, it is our assumption that asymptomatic transmission was not considered, as it was only articulated in one protocol.

Throughout all 13 protocols the supporting information varied, with six citing the WHO, 10 referring to their respective provincial chapter of the CDC or government health officer/ministry, and three mentioning their corresponding WorkSafe^®^ chapter. In addition, the recommendations seemed to be based on a very limited number of referenced publications, or none at all; regardless, the messages primarily remained clear and were either delivered concisely or extensively, with protocols ranging between 7 and 60 pages in length. Guidelines and policy recommendations, including protocols, should be grounded in evidence-based practice approaches and in precautionary principles, as suggested by Crosby and Crosby [63]. Without more evidence-based information on SARS-CoV-2 and oral health care, future studies should focus on the hard evidence that does exist and can reasonably be extrapolated to the provision of oral health care services; they should also include providers’ perspectives to elicit their views on the future of delivering oral health care during the COVID-19 pandemic. As the epidemiology of SARS-CoV-2 evolves, we should be able to develop a better understanding of the actual risks that AGPs pose to our patients, to staff, and to the public. Different levels of SARS-CoV-2 infection rates and of COVID-19 development were observed across Canada; however, the extent to which such variation influenced the content of the protocols presented herein is unknown, as is the effect of this variation on the dissimilarities across some of their recommendations. Moreover, consensus may take decades, particularly considering COVID-19 is a new disease that is still evolving; the scientific evidence we have thus far is not dogma, and our views need to be modified as new knowledge is produced and new experiences are presented.

Our study is not without limitations. By focusing only on the restructuring of general dentistry practice, protocols aimed at restructuring the practice of dental hygienists, dental therapists, dental technicians, denturists, and dental specialties were excluded; we do recognize the recent debates around the differences in guidelines issued by different regulatory bodies, and the significant challenges that such differences may cause for those clinics in which dentists and dental hygienists and other providers work together. Future studies should include protocols that deal with the practice of allied oral health care services and the dental specialties during a pandemic. Its sole focus on Canada also excluded international protocols; although, we strongly believe that the evidence-base behind the recommendations of these protocols are indeed applicable to other countries given the universality of dental care and the impact of the COVID-19 pandemic. That is, the evidence behind a given recommendation (e.g., the use of specific masks, or the office layout) is available to better inform practitioners and researchers, regardless of where they are located. We did not assess protocols that were updated after the date shown in Table 1. Given the constant flow of new information around COVID-19, we may have missed protocols that contain new or more evidence-based data. Although the research team assessed each protocol using the form and met to discuss the findings, some of the information presented in Table 1 might have been misinterpreted. This is a possibility due to the contradictory information within some protocols and the ambiguous and open-for-interpretation information in others. Follow-up studies should focus on the extent to which such protocols were effective in curtailing the rate of SARS-CoV-2 infections within a population, and within the dental providers and staff themselves. Lastly, future studies should also focus on the knowledge dental health care personnel hold in understanding the implications of potential transmission of the SARS-CoV-2 virus in a dental clinic setting.

## Conclusions

The constantly evolving knowledge and epidemiology around SARS-CoV-2 poses challenges to the development of protocols aimed at reopening and restructuring the delivery of oral health care in Canada and elsewhere; such challenges are even more worrisome during the second wave of even more severe infections underway in many countries, including Canada. The 13 protocols reviewed in this study aimed at limiting the transmission of the virus and were assessed in eight areas, from personal protective equipment to COVID-19 screening measures. Although all protocols seemed easy to read, it was apparent that good quality evidence-based information was limited, and non-referenced material was used. As new evidence-based knowledge is produced, these protocols and recommendations will need to continue to be updated and made available to the profession and their regulators so that operative policies are continuously introduced to better guide the dental profession. Dental providers and their staff need to keep themselves updated with new information regarding COVID-19 and the provision of oral health care for the safety of their patients and the community.

## Data Availability

The 13 protocols with data that supported the findings of this study are available from the Office of the Chief Dental Officer of Canada, at https://www.diac.ca/wp-content/uploads/2020/05/National-Reopen-Strategy-.pdf, last updated on May 22 2020.

## Abbreviations

AGP: Aerosol generating procedure
ASTM: American Society for Testing and Materials
BC: British Columbia
CDC: Centers for Disease Control and Prevention
COVID-19: Coronavirus disease 2019
HIV: Human immunodeficiency virus
HO_2_: Hydroperoxyl radical
HVAC: Heating, ventilation, and air conditioning
NAGP: Non-aerosol generating procedure
PCR: Polymerase chain reaction
PPE: Personal protective equipment
RNA: Ribonucleic acid
SARS-CoV-2: Severe acute respiratory syndrome coronavirus 2
UPs: Universal precautions
WHO: World Health Organization
